# Supplementary Motor Area During Motor Actions in Athletes With Neurological Disorders: Observational Study of Cases and Controls

**DOI:** 10.1002/brb3.71522

**Published:** 2026-06-04

**Authors:** Francisco Echevarría‐Lasaga, Ismael Sanz‐Esteban, Cecilia Estrada‐Barranco, Isabel Rodríguez‐Costa, Juan José García‐Hernández, José Ignacio Serrano

**Affiliations:** ^1^ Department of Physiotherapy, Faculty of Sport Sciences, Neurosciences and Physical Therapy Research Group Universidad Europea de Madrid Villaviciosa De Odón Spain; ^2^ Facultad De Medicina y Ciencias De La Salud Unidad Docente de Fisioterapia Alcalá de Henares Spain; ^3^ Segunda Parte Foundation, Madrid, Spain; Faculty of Health Sciences, Exercise and Sport Sciences University of Francisco de Vitoria Pozuelo de Alarcón Spain; ^4^ Computational Modeling of Intelligence Group, Center for Automation and Robotics (CAR), CSIC‐UPM Arganda del Rey Spain

**Keywords:** adaptive sport, electroencephalography, motor area, motor planning, neurological disorders, supplementary

## Abstract

**Objective:**

To determine differences in the activation of the supplementary motor area (SMA) during the motor preparation and anticipatory phase in athletes with and without neurological disorders during the action of kicking a ball in static and dynamic conditions.

**Methods:**

Two groups of 15 athletes each (disabled and non‐disabled) were recruited. Surface electroencephalography (EEG) was used to record cortical activity while participants performed two motor tasks. Prior to each trial, participants remained with their eyes closed for 20 s to ensure a neutral baseline state. EEG analyses were conducted during the period from eyes opening to ball contact. Cortical source density was extracted from the EEG signal and compared between the two groups and tasks across different frequency bands.

**Results:**

Significantly higher activation of the SMA and premotor areas activity was found in the experimental group versus the control group during the preparatory phase still target task with the non‐dominant/more affected leg. In reaction time terms, significant differences between groups were found during the moving target task with the non‐dominant/more affected leg and during the still target task with the dominant/less‐affected leg.

**Conclusions:**

The differences observed in SMA activation suggest possible variations in motor preparation processes among the groups; these findings indicate the presence of compensatory neural strategies that may facilitate motor control in athletes with neurological disorders. These results may serve as a basis for future research on adapted sports training and neurorehabilitation.

**Significance:**

People with neurological disorders may exhibit a series of motor deficiencies that affect sports practice. Delving into the mechanisms involved can improve performance and motor planning in other daily‐living functions.

## Graphical abstract

Athletes with neurological disorders showed increased supplementary motor and premotor area activation during soccer‐specific motor preparation with respect to healthy controls, particularly with the affected limb, suggesting compensatory neural strategies that support motor control and performance. These findings highlight the role of neuroplastic adaptations in adapted sport and neurorehabilitation.

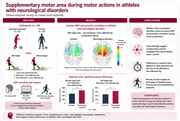

## 1 Introduction

Neurological disorders constitute one of the leading causes of disability worldwide (Feigin et al. 2024). Among them, motor neurological impairments, cerebral palsy, acquired brain injury, and hereditary motor disorders represent a high proportion of long‐term disability. Cerebral palsy is the most common cause of motor disability in childhood and has an estimated prevalence of approximately 2–3 cases per 1000 live births in Europe. In Spain, the reported estimates are consistent with these data (Badia et al. 2013). Furthermore, acquired brain injury represents another major source of neurological disability, with incidence rates reaching 191 cases per 100,000 inhabitants in some Spanish populations. In addition, rare neurodegenerative motor disorders, such as ataxias and hereditary spastic paraplegia, further increase—although with lower prevalence—the number of neurological disability cases in Spain (Ortega‐Suero et al. 2023).

Currently, rehabilitation programs increasingly incorporate physical activity. There is solid evidence that training and the acquisition of physical skills can induce changes in neural activation, particularly during the execution of functional skills (Hill and Schneider 2006; Wright et al. 2010). As a result of this integration, a growing number of programs have been developed to adapt and include sports practice within neurorehabilitation processes, as they provide significant musculoskeletal, neural, cardiorespiratory, and cognitive benefits (Pérez‐Tejero et al. 2012; Hanrahan 2015; Turner‐Stokes et al. 2008).

Sports practice requires the execution of precise and coordinated technical actions. In sports such as football, successful performance depends on efficient motor planning, understood as the process of anticipating and organizing movement sequences prior to their voluntary execution. Performance in this discipline is determined not only by technical skill but also by processing speed and movement regulation, processes supported by the basal ganglia–thalamo–cortical circuit (Takakusaki 2017; Rodríguez‐Bazurto and Aguilar‐Morocho 2021). Within this circuit, structures such as the thalamus, basal ganglia, and the dorsal premotor area work together to regulate movement speed and accuracy during motor reaching tasks (Shirinbayan et al. 2019).

However, in athletes with neurological disorders such as cerebral palsy or hemiparesis, this motor control system faces significant structural and functional challenges that may compromise the effectiveness of the motor gesture (Dinomais et al. 2014; Liu et al. 2020). Neurological motor disorders, including cerebral palsy, stroke, traumatic brain injury, and other central nervous system lesions, share common alterations in motor planning networks involving structures such as the premotor areas and the supplementary motor area (Nachev et al. 2008).

At the cortical level, the Supplementary Motor Area (SMA), corresponding to Brodmann area 6, represents a central hub for complex motor planning (Boy et al. 2010; Cañas et al. 2018). In electrophysiological studies, motor planning processes in this region have also been associated with oscillatory activity in the alpha and beta frequency bands, which are thought to reflect the selection and preparation of motor programs within sensorimotor networks. Located in the medial portion of the superior frontal gyrus, the SMA is involved in the preparation, initiation, and execution of voluntary movements, as well as in the unconscious inhibition of automatic motor responses (Boy et al. 2010). A critical feature of this region is its extensive interhemispheric connectivity, as it is one of the cortical areas with the highest number of transcallosal connections, making it essential for the coordination of lower‐limb movements (Ruddy et al. 2017; Sahyoun et al. 2004; Newton et al. 2008). It is estimated that approximately ten percent of the afferent information of corticospinal tracts originates in the SMA, highlighting its relevance in descending motor control (Potgieser et al. 2014).

Despite the importance of these structures, a critical gap remains in the scientific literature regarding motor control in athletes with specific neurological disorders during real sports tasks. Although it has been demonstrated that the acquisition of expert skills can induce changes in neural activation and improve cortical efficiency—a phenomenon known as “neural efficiency”—most of these studies have focused on populations without disabilities (Hill and Schneider 2006; Wright et al. 2010; Zhang et al. 2019; Yin et al. 2025). In contrast, studies conducted in clinical contexts suggest that brain lesions may lead to increased SMA activation as a compensatory mechanism to respond to functional imbalance (Eckert et al. 2006; Foltys et al. 2003; Caçola et al. 2016). However, it remains unclear whether these compensatory mechanisms manifest in the same way in athletes with motor disabilities during the execution of specialized technical actions such as ball kicking.

Movement speed constitutes a key element for the efficient execution of any motor action; therefore, its appropriate neural regulation is essential (Shirinbayan et al. 2019). The most recent studies on reaction speed have used neuroimaging techniques, particularly functional magnetic resonance imaging (fMRI), showing that the contralateral dorsal premotor area, the SMA, the primary motor cortex (M1), the thalamus, and the basal ganglia are associated with movement processing speed and execution. These findings suggest the existence of a basal ganglia–thalamo–cortical circuit involved in the regulation of motor speed (Shirinbayan et al. 2019).

Furthermore, the relationship between motor planning and hemispheric specialization represents a determining factor. Traditionally, the left hemisphere has been proposed to be specialized in motor planning and coordination, whereas the right hemisphere plays a key role in modifying ongoing actions in response to external stimuli (Mutha et al. 2012). In individuals with neurological damage, this lateralization is often altered, showing bilateral activation patterns that may reflect a greater demand for cortical resources to compensate for the loss of functional specificity (Dinomais et al. 2014; Liu et al. 2020). This increased load in motor planning processes is associated with reduced processing speed and longer reaction times compared with individuals without neurological impairment (Welniarz et al. 2019; Churchill et al. 2021). In this sense, activity within motor planning regions such as the SMA may influence the efficiency with which motor commands are prepared and initiated, ultimately affecting reaction time during goal‐directed motor actions. In this regard, Welniarz et al. (2019) conducted a study in which reaction time was measured during different movements in a control group without neurological impairment and an experimental group with neurological impairment. The results showed that the control group presented significantly shorter reaction times compared with the experimental group.

Further investigation into the specific mechanisms of motor planning and execution in athletes with neurological disabilities may provide valuable information for rehabilitation and the design of adapted training programs. Such knowledge could optimize training strategies, improve sports performance, and promote the inclusion of these individuals in the sporting environment (Pérez‐Tejero et al. 2012; Hanrahan 2015; Rodríguez‐Bazurto and Aguilar‐Morocho 2021).

Previous studies in clinical populations have reported increased recruitment of the supplementary motor area during voluntary movement following neurological injury, interpreted as a compensatory mechanism supporting motor planning (Foltys et al. 2003; Caçola et al. 2016). In addition, neurological impairment has been associated with slower processing speed and longer reaction times during motor tasks (Welniarz et al. 2019; Churchill et al. 2021). In this context, athletes with neurological disorders may exhibit different patterns of SMA activation and reaction times compared with athletes without neurological impairment during sport‐specific motor actions.

Based on the above, the aim of the present study is to determine whether significant differences exist in SMA activation between athletes with neurological disorders and athletes without disabilities during ball‐kicking tasks under static and dynamic conditions.

## 2 Methods

This is an analytical cross‐sectional observational study with a control group on brain activity in subjects with cerebral alterations who practice soccer. A sample of 30 athletes divided into two groups, 15 with cerebral alterations and 15 without, was obtained to measure brain activity during the action of kicking a ball in a bipedal position, both static and in motion. An electroencephalogram (EEG) was used to measure cortical activity.

The sample size of 15 participants per group was calculated using the GRANMO program, accepting an alpha risk of 0.05 and a beta risk of 0.2 in a bilateral contrast. The calculation was based on the primary outcome of the study, defined as SMA source density during the preparatory phase of the motor task.

The experimental group consisted of athletes with various neurological conditions (cerebral palsy, stroke, traumatic brain injury, tumor, hypoxia, and tetraparesis). Although these conditions differ in terms of lesion location and pathophysiology, they all shared a common characteristic: a neurological disability that affected their motor control, which plays an active role in adapted soccer training. Since the sample size was small, it was not possible to conduct analyses by subgroup.

Individuals aged 18 to 50, with a prior diagnosis of a neurological disorder (experimental group) or without any diagnosis (control group), who practiced soccer at least twice a week, were included. Individuals with insufficient cognitive capacity, absolute contraindications for the tests, and visual impairment hindering the execution of motor acts in the study were excluded. Ethical approval was obtained from the Hospital Clínico San Carlos and the European University.

Recruitment, data collection, and testing were conducted between May and June 2023, in sessions of approximately ten minutes. Demographic data were collected, and an electroencephalographic cap was fitted to the athletes.

The sample was a convenience sample derived from obtaining participants with neurological or brain impairment who play football at the Second Part Foundation.

**FIGURE 1:**
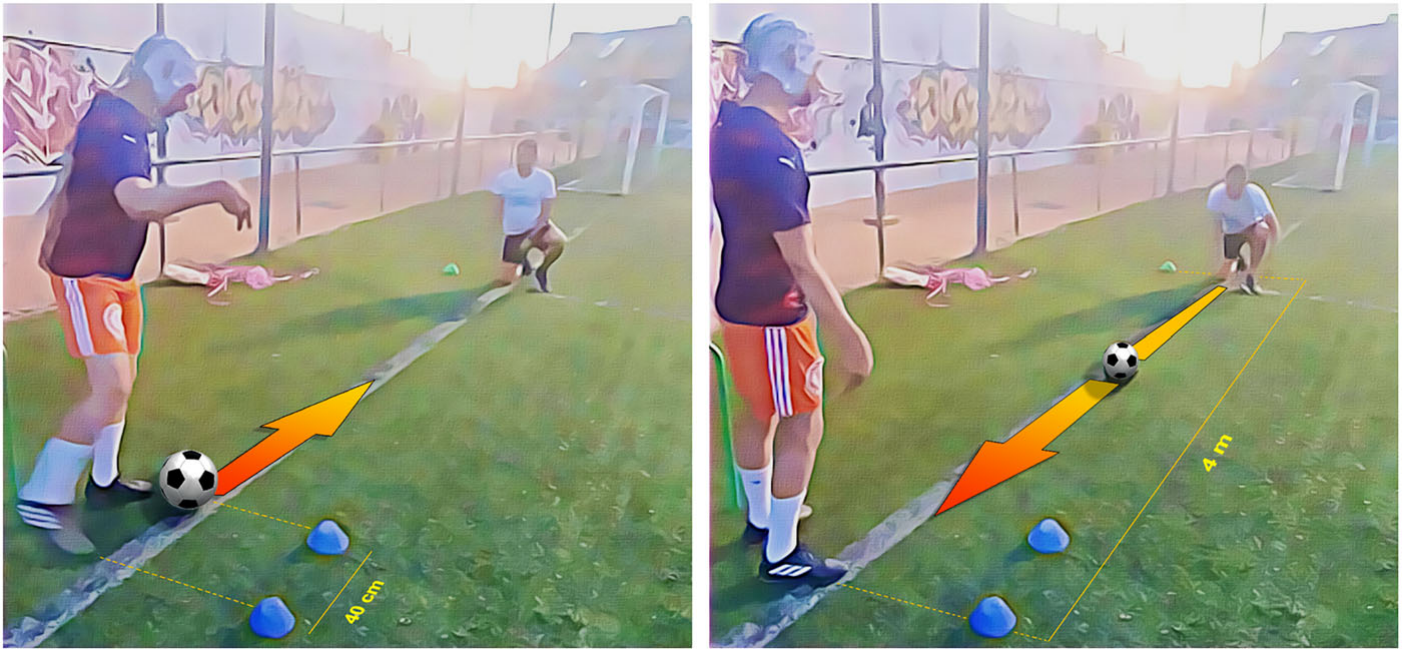
Experimental setup and tasks. Left: Still target task. The participants kicked the stopped ball forward toward the researcher after 20 s of standing with their eyes closed. Right: Moving target task. After 20 s of standing with their eyes closed, the researcher threw the ball low to the participants, and they kicked it back to him.

Participants performed two tasks: the static target task and the moving target task (Figure 1). Prior to each trial, participants were informed about the type of task and the leg to be used (healthy participants always started with their non‐dominant leg, while participants with neurological impairments always started with their affected leg, regardless of its dominance). Participants in both groups stood with their eyes closed for 20 s before performing the kick. This period was used to allow participants to reach an initial neutral state and to minimize prior visual information or anticipatory motor processes as much as possible. During this period, participants were instructed to remain relaxed and breathe calmly. It is important to note that this phase was not considered part of the motor planning process and was not included in the EEG analyses. At the end of this period, a standardized verbal cue (“Open your eyes and kick the ball”) was given to initiate the movement. This instruction was the same for all participants. In the static target task, they kicked a ball placed 40 cm in front of them toward the therapist, first using the non‐dominant/most affected leg and then the other, after standing for 20 s with their eyes closed. In the moving target task, they kicked a ball coming from the researcher positioned 4 meters in front of them, also starting with the non‐dominant/most affected leg and then the other, after standing for 20 s with their eyes closed. Each activity was performed twice, once with each leg. The terms used in this study distinguish between the dominant leg and neurological impairment. The “affected leg” is the limb affected by a neurological condition, regardless of its dominance. The “unaffected leg” is the limb that is not affected. The terms “dominant” and “non‐dominant” refer to the leg the participant prefers to use for kicking. The analyses take both factors into account to allow for accurate comparisons, even when the dominant leg is the affected one. EEG activity was recorded continuously throughout the task; however, only the time window between eyes opening and ball contact was included in the analyses. EEG source density in the SMA and premotor area, together with reaction times from the eyes opening to ball were considered the outcome measures.

### 2.1 EEG Processing

The electroencephalographic activity was acquired and digitized by a Brainvison actiCHAmp amplifier (Brain Products GmbH, Germany) with 32 active actiCAP slim Ag/Cl electrodes placed in the scalp at Fp1, Fp2, AF3, AF4, F7, F3, Fz, F4, F8, FC5, FC1, FC2, FC6, T7, C3, Cz, C4, T8, CP5, CP1, CP2, CP6, P7, P3, Pz, P4, P8, PO3, PO4, O1, Oz, and O2 locations according to the 10–20 system. The signal was recorded at 512 Hz with the ground reference at the AFz location.

The EEG was continuously registered for each subject, task, and leg individually, resulting in a file for each of the former combinations. Each file was processed as follows. The signal was first resampled to 256 Hz. Then, noise was removed by the Artifact Subspace Reconstruction (ASR) algorithm (Chang et al. 2020), with 0.25–0.75 boundaries for high‐pass filtering and 20 as the threshold criterion. After that, the signal was bandpass filtered between 3 and 31 Hz. Next, the independent components were extracted with the ICA algorithm, and the ICLabel algorithm (Pion‐Tonachini et al. 2019) was used to identify and subtract the eye‐ and muscle‐related artifacts, with a minimum confidence threshold of 0.7. The signal was then average referenced, and the channels whose spectrum deviated more than 2 times the standard deviation were interpolated by spherical spline interpolation (Ferree, 2006). Finally, the time of the kick was determined as the average time to the first signal burst after opening the eyes, given that the kick produced a global artifact that is not removed by the previous steps. The signal between the eyes opening and the kick was kept for further source analysis. All the mentioned signal processing procedures were performed by a script using MATLAB v2017b (Mathworks Inc., USA) and the EEGLAB v2023.0 toolbox (Delorme and Makeig 2004). The implementations used for the mentioned algorithms are the ones included in the indicated EEGLAb version. All the parameters not explicitly referenced above were set as their default values according to the EEGLAb implementations.

**FIGURE 2:**
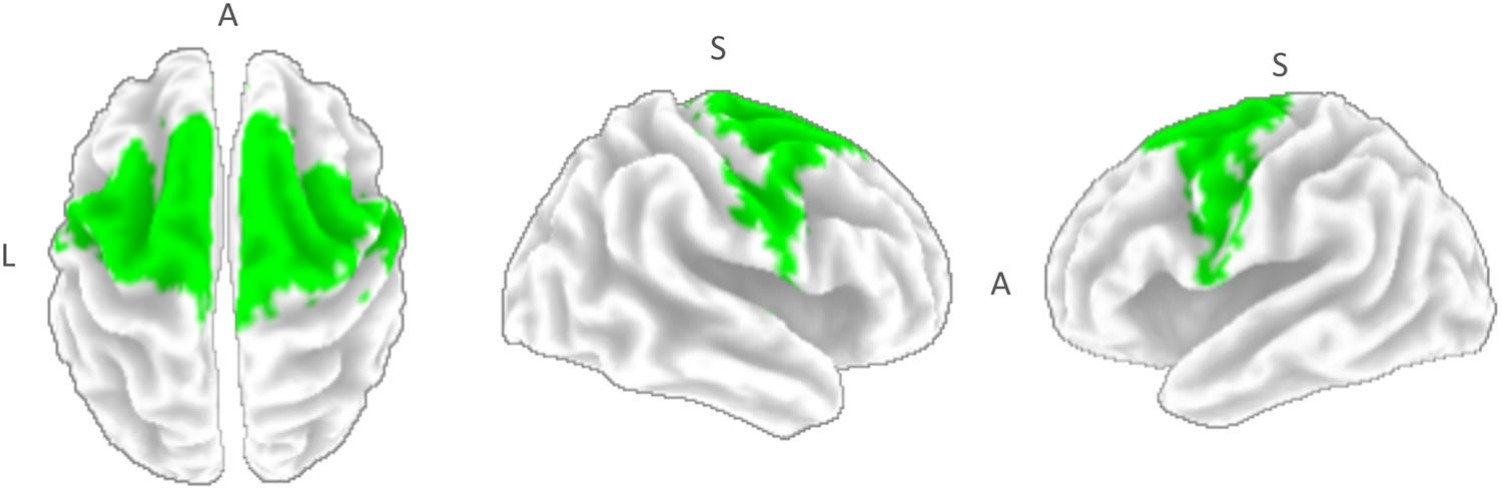
Cortical area (green) corresponding to the PA and SMA (BA 6) as used in this study in an inflated brain model. Abbreviations: A, anterior; L, left; S, superior.

The LORETA‐Key v20220902 software (KEY Institute for Brain‐Mind Research, University of Zurich, Switzerland) was used to extract the cortical source densities from the processed EEG intervals by using the eLORETA source localization algorithm (Pascual‐Marqui et al. 2011) in six different frequency bands: theta (4 Hz–7 Hz), low alpha (7 Hz–10 Hz), high alpha (10 Hz–13 Hz), low beta (13 Hz–18 Hz), mid beta (18 Hz–21 Hz), and high beta (21 Hz–30 Hz). The eLORETA algorithm estimates the source density in each of the 6239 voxels (cubes) at 5 mm spatial resolution, conforming to the intracerebral volume. The average source density from the voxels that belong to the Brodmann area 6 (PA and SMA, Figure 2) of both hemispheres combined as a single ROI was computed for each frequency band and compared between groups.

There are several reasons to consider the bilateral SMA as a single ROI. First, the SMA has the most extensive interhemispheric connectivity among all cortical regions (Ruddy et al., 2017). Moreover, the literature has already evidenced the bilateral implication of the SMA in lower limb and kicking movements (Sahyoun et al., 2004; Newton et al., 2008; Yin et al., 2025), as well as in locomotion‐related tasks (Jaeger et al., 2014). In addition, bilateral SMA dysfunction is a convergent neuropathological feature across stroke (Liu et al. 2020) and cerebral palsy (Dinomais et al. 2014), conditions predominant in the study population. It has also been evidenced that expertise‐related SMA changes, such as the ones observed in athletes, are present bilaterally (Zhang et al. 2019). Finally, the lateralization that has been observed in the literature is primarily for fine, distal, and unimanual hand movements (Kapreli et al. 2006). In summary, lower‐limb representations are significantly more bilateral than upper‐limb representations; the strong homotopic connectivity between bilateral SMA means that even when activation differences exist (Mancuso et al. 2019), the two sides are tightly functionally coupled; in motor disorders, SMA changes are characteristically bilateral, making hemispheric separation less informative; and finally, in a study involving patients with motor disorders of different etiology—some with unilateral lesions, some with bilateral or diffuse pathology, and different dominant sides—lateralization patterns will vary unpredictably across patients, and a bilateral ROI captures the functionally meaningful signal common to all participants.

The EEG processing was performed without group distinction by a researcher blind to the data acquisition.

### 2.2 Data Analysis and Statistics

Differences in demographic and anthropometric numerical variables between groups were tested by ANOVA after confirmation of normality by the Shapiro–Wilk test and Q–Q plots. The differences in the distributions of demographic and anthropometric categorical variables were tested by the chi‐square test.

Since almost all reaction time and SMA activation distributions in all frequency bands were far from normal (Shapiro–Wilk test and Q–Q plots) in subsets of legs and tasks for all groups, the differences between groups were tested by the Kruskal–Wallis test. Ninety‐nine percent confidence intervals for *p*‐values (C.I. (*p*)) from Monte Carlo estimations, as well as the effect size epsilon‐squared (𝜖^2^), are reported for the significant or nearly significant differences. The differences within groups between legs or tasks were tested by the Wilcoxon signed‐rank test for related measures. In this latter case, the effect size *r* is reported.

All the above‐mentioned analyses were performed with IBM SPSS Statistics for Windows v29 software (Armonk, NY: IBM Corp.).

The data analysis was performed by a researcher blind to the data acquisition process and the nature of groups.

## 3 Results

All individuals potentially eligible, confirmed, and included in the study completed the measurements and were analyzed. The final sample consisted of a total of 30 individuals, 3 females (Table 1).

**TABLE 1:** Demographic, anthropometric, and clinical variables for each group.

Avg. (std.)/% (#)		Experimental (*N* = 15)	Control (*N* = 15)	Statistic
Age (years)		27.6 (7.8)	26.2 (7.5)	*F*(1, 28) = 0.032, *p* = 0.859
Women		0.00% (0)	20.00% (2)	*χ* ^2^(1) = 1.481, *p* = 0.224
Dominant/Less affected leg Right		53.33% (8)	100.00% (15)	*χ* ^2^(1) = 6.708, *p* = 0.010
Height (cm)		1.70 (.071)	1.73 (.105)	*F*(1, 28) = 0.951, *p* = 0.338
Weight (Kg)		67.69 (12.68)	68.92 (11.41)	*F*(1, 28) = 0.092, *p* = 0.764
BMI		23.4 (3.66)	22.82 (2.63)	*F*(1, 28) = 0.094, *p* = 0.761
Motor disorder	Ataxia	20.00% (3)		
	Dyskinesia	6.67% (1)		
	Dysparesis	6.67% (1)		
	L Hemiparesis	13.33% (2)		
	R Hemiparesis	33.33% (5)		
	Tetraparesis	20.00% (3)		
	Anoxia	6.67% (1)		
Etiology	Cerebral palsy	53.33% (8)		
	Hydrocephalus	6.67% (1)		
	Stroke	13.33% (2)		
	TBI	13.33% (2)		
	Brain tumor	6.67% (1)		

Abbreviations: BMI, Body‐Mass Index; L, left; R, right.

Data from one of the participants in the experimental group during the still‐target task were discarded due to recording errors. No significant differences in age, sex, height, weight, and BMI were found. However, while all participants in the control group were right‐legged, the less affected leg in the experimental group was the right one in over 50% of the participants. A heterogeneity of motor disorders and etiologies was present in the experimental group, hemiparesis and cerebral palsy being predominant, respectively.

### 3.1 Differences of Activation of the Supplementary Motor and Premotor Areas Between Groups

**FIGURE 3:**
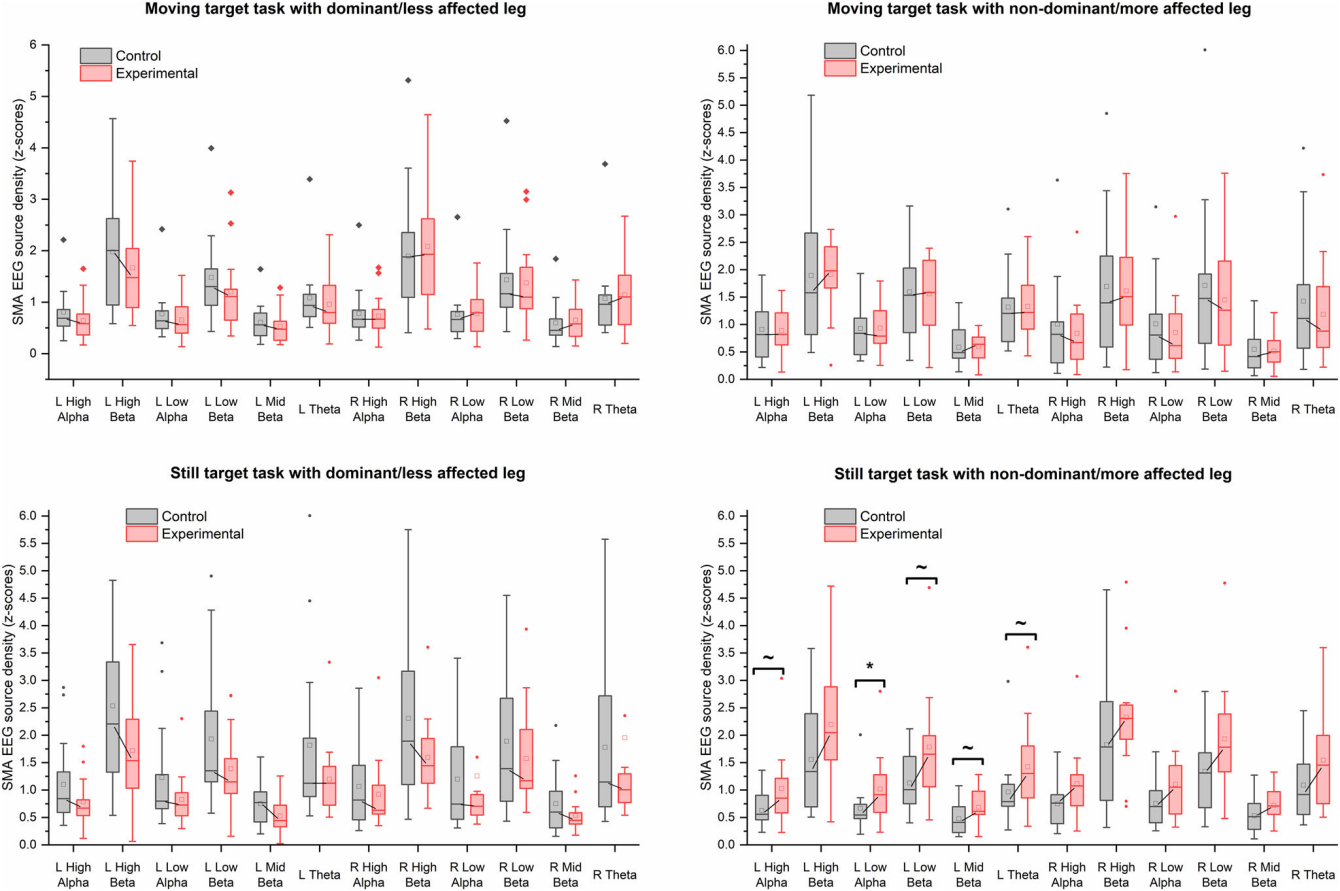
Boxplots of the SMA‐normalized source density during the moving target (top row) and still target (bottom row) tasks with dominant/less affected (left column) and non‐dominant/more affected (right column) legs for each group (colors) in all frequency bands (x‐axis). Squares within the boxes represent the median values. Bars with asterisks denote statistically significant differences between groups. Bars with diacritical marks denote a trend toward significant differences (*p* < 0.08) between groups.

**FIGURE 4:**
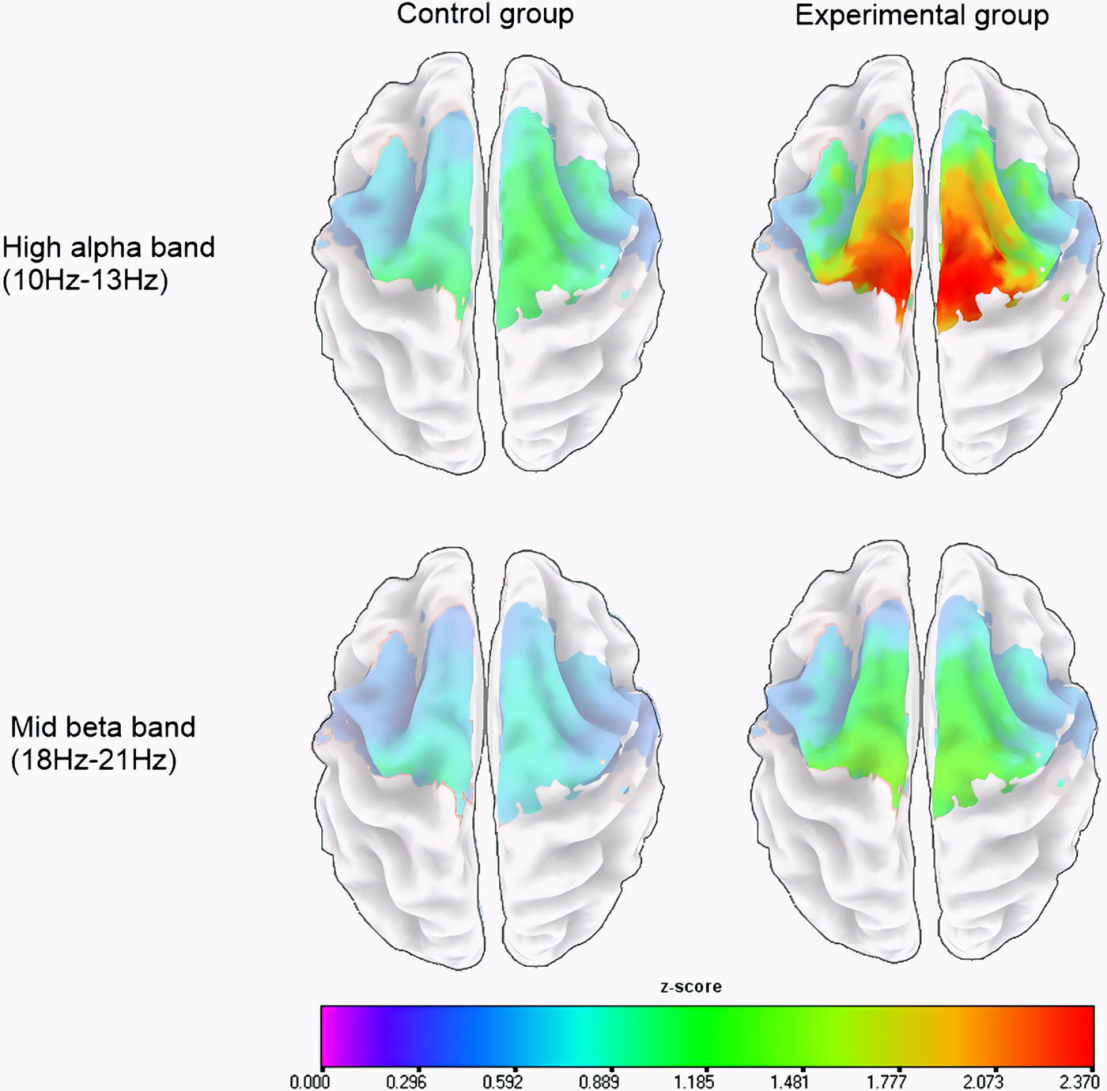
Cortical maps of source density (average *z*‐values) of the control and experimental groups during the still target task with the non‐dominant/more affected leg in the frequency bands that presented a significant statistical difference between groups.

The only statistically significant results were found during the still target task with the non‐dominant/more affected leg (Figure 3). In this case, the participants in the experimental group showed a significantly higher activation of the SMA than the control group in the high alpha (2.14 ± 1.33 vs. 1.37 ± 0.60, H(29) = 4.030, *p* = 0.045, C.I.(*p*) = (0.037–0.047), 𝜖^2^ = 0.144) and mid beta (1.41 ± 0.57 vs. 1.01 ± 0.60, H(29) = 3.857, *p* = 0.050, C.I.(*p*) = (0.042–0.053), 𝜖^2^ = 0.138) frequency bands (Figure 4) and a nearly significant higher activation in the low beta (3.72 ± 0.203 vs. 2.47 ± 1.10, H(29) = 3.522, *p* = 0.061, C.I.(*p*) = (0.053–0.065), 𝜖^2^ = 0.126) and high beta (4.52 ± 2.01 vs. 3.39 ± 2.13, H(29) = 3.360, *p* = 0.067, C.I.(*p*) = (0.059–0.072), 𝜖^2^ = 0.120) frequency bands. The opposite results (higher SMA activation in the control group) are present in the moving target task, but no significant difference was found in this case. All the effect sizes reported are moderate to large.

With the dominant/less affected leg, the control group presents a higher activation of the SMA overall, regardless of the task, although these results lack statistical significance.

### 3.2 Differences of Activation of the Supplementary Motor and Premotor Areas Between Legs

**FIGURE 5:**
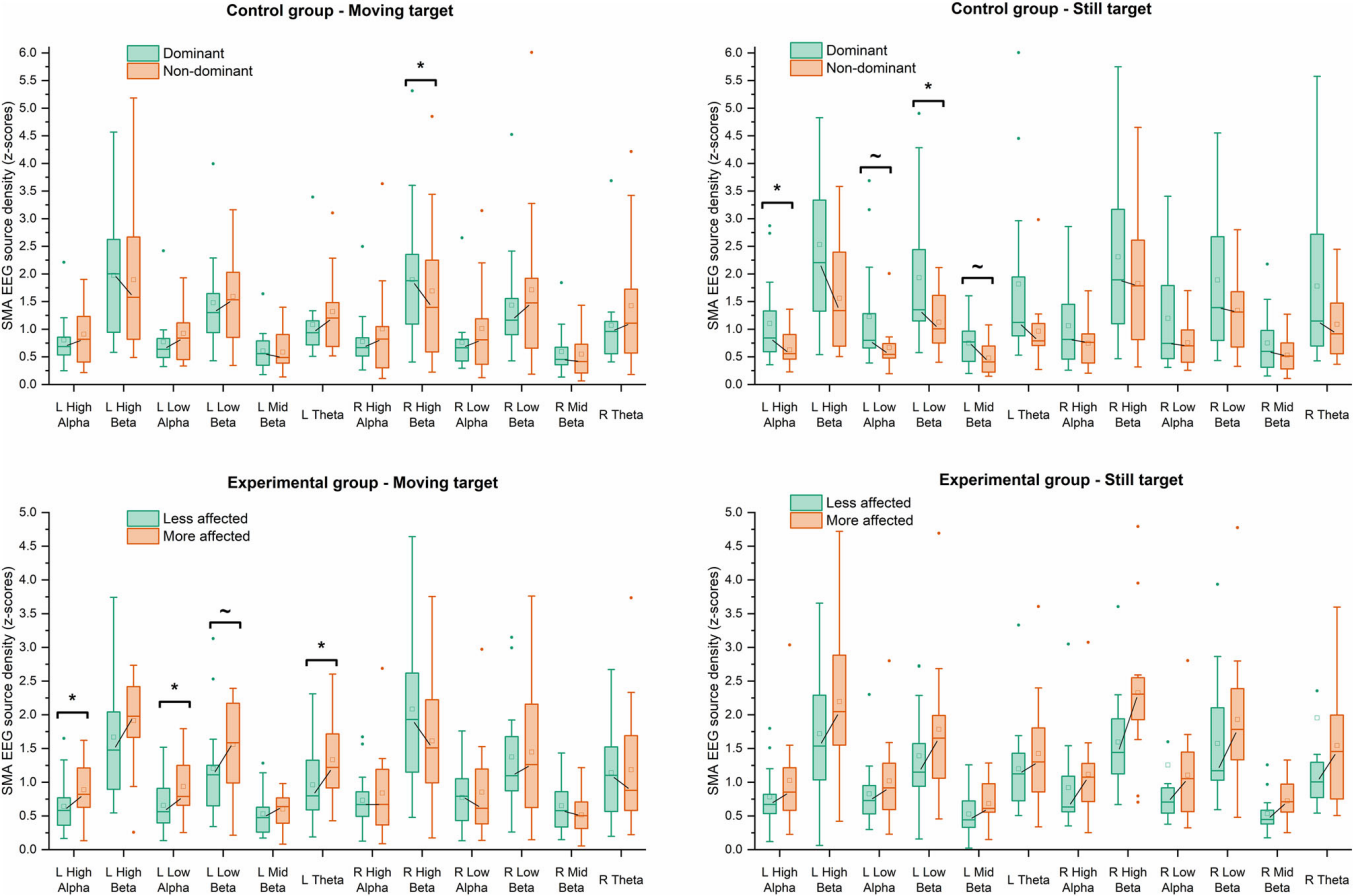
Boxplots of the SMA‐normalized source density for the two legs (colors) during the moving target (left column) and still target (right column) tasks for the control (top row) and experimental (bottom rows) groups in all frequency bands. Squares within the boxes represent the median values. Bars with asterisks denote statistically significant differences between legs. Bars with diacritical marks denote a trend toward significant differences (*p* < 0.08) between legs.

Figure 5 shows the boxplots of the normalized SMA activation between legs for each group and task.

With respect to the control group, the participants showed an overall greater activation of the SMA with the dominant leg during the still target task, showing only nearly statistically significant differences between legs in the low beta band (3.82 ± 2.55 for the dominant leg vs. 2.47 ± 1.10 for the non‐dominant leg, *Z* = 29.000, *p* = 0.078, *r* = 0.472).

**FIGURE 6:**
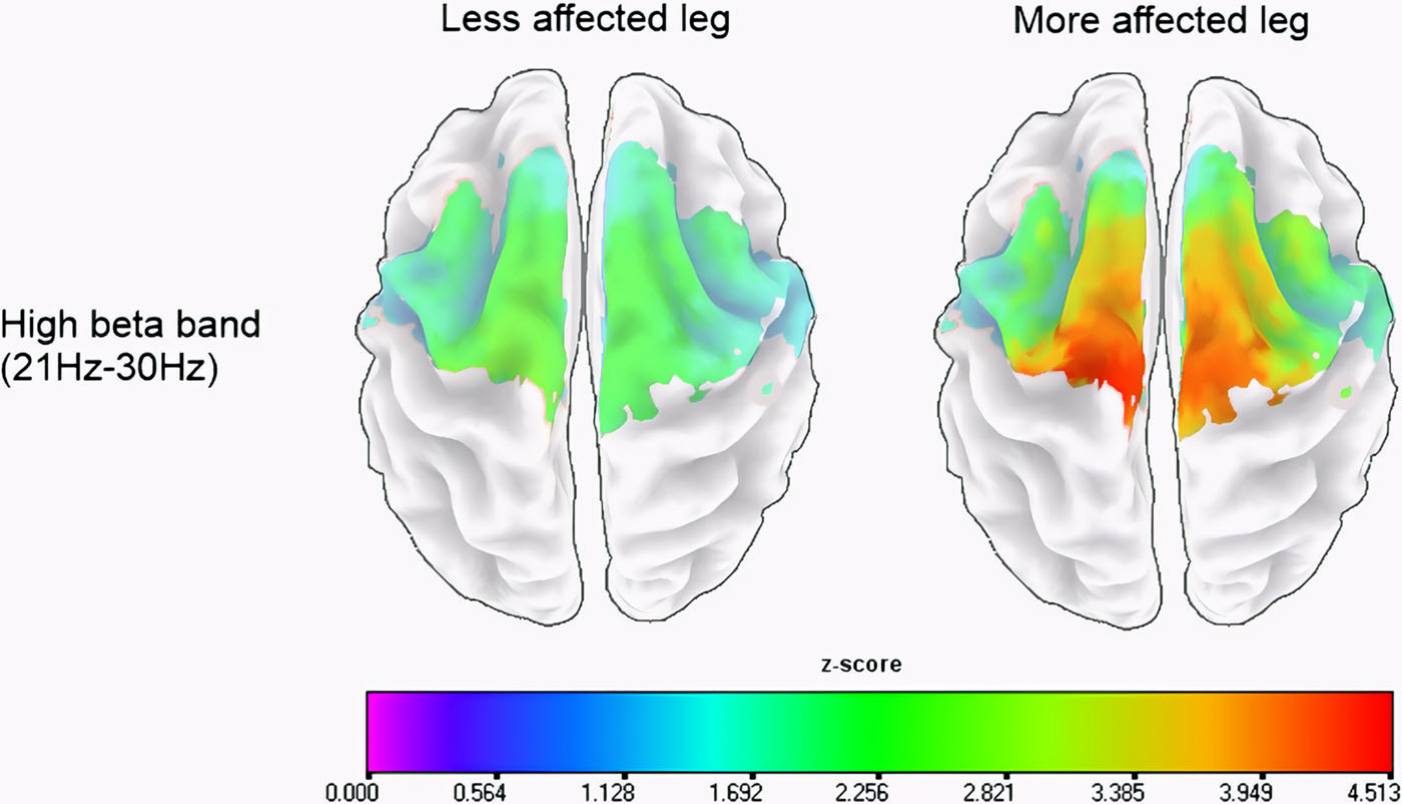
Cortical maps of source density (average *z*‐values) in the experimental group during the still target task for the less affected and more affected legs in the frequency band that presented a significant statistical difference between sides.

Regarding the experimental group, the only significant difference was found in the still target task. Concretely, the more affected leg showed a significantly higher activation of the SMA than the less affected leg in the high beta band (4.52 ± 2.01 vs. 3.31 ± 1.56, *Z* = 84.000, *p* = 0.048, *r* = 0.528) (Figure 6). Similarly, the mid beta band showed a nearly significant difference (1.41 ± 0.57 vs. 1.06 ± 0.53, *Z* = 82.000, *p* = 0.064, *r* = 0.495). All the reported effect sizes are moderate to large.

### 3.3 Differences of Activation of the Supplementary Motor and Premotor Areas Between Tasks

**FIGURE 7:**
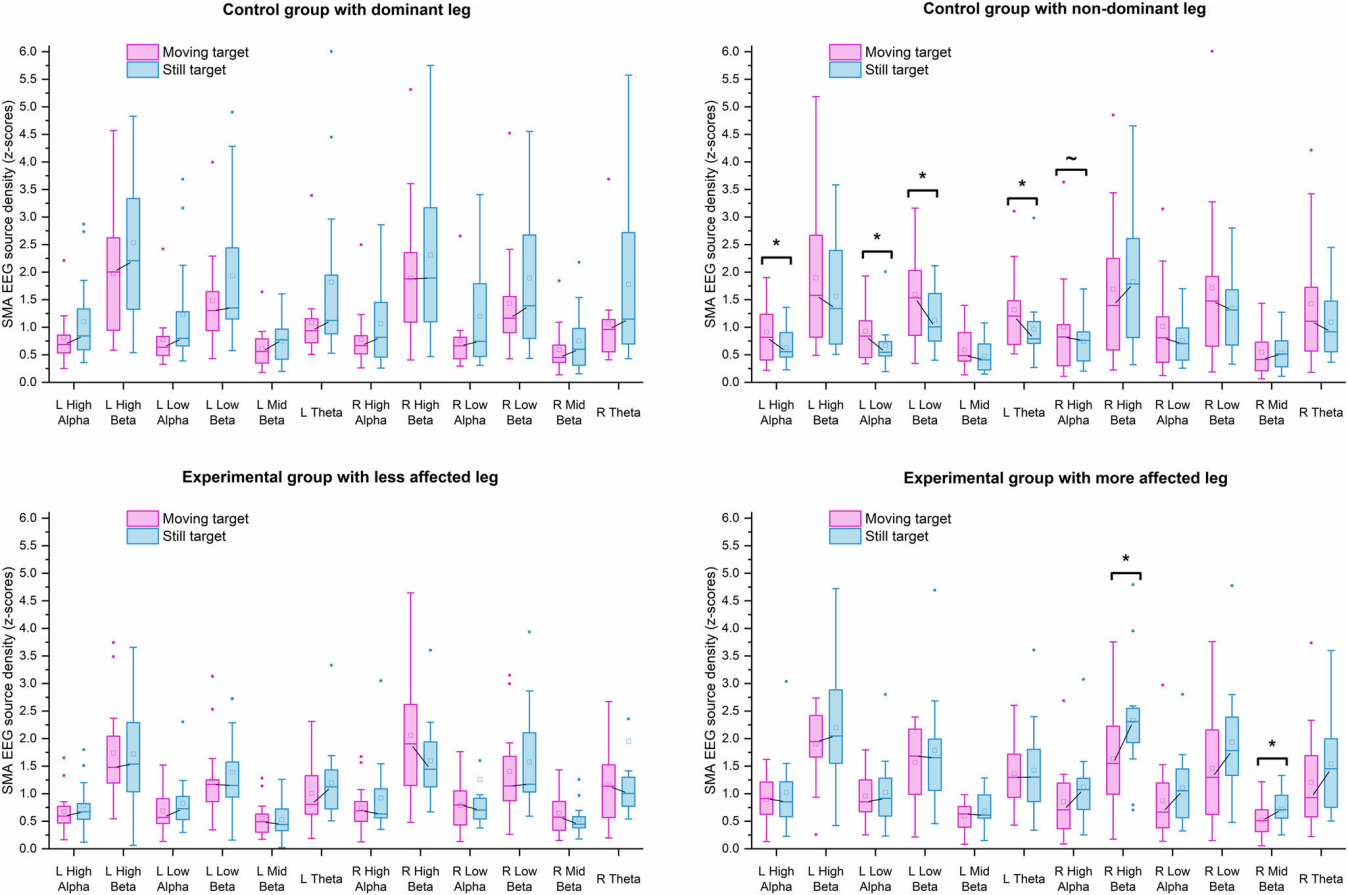
Boxplots of the SMA‐normalized source density for the two tasks (colors), with the dominant/less affected (left column) and non‐dominant/more affected (right column) legs for the control (top row) and experimental (bottom row) groups, in all frequency bands. Squares within the boxes represent the median values. Bars with asterisks denote statistically significant differences between tasks. Bars with diacritical marks denote a trend toward significant differences (*p* < 0.08) between tasks.

Figure 7 shows the boxplots of the normalized SMA activation between tasks for each group and leg.

**FIGURE 8:**
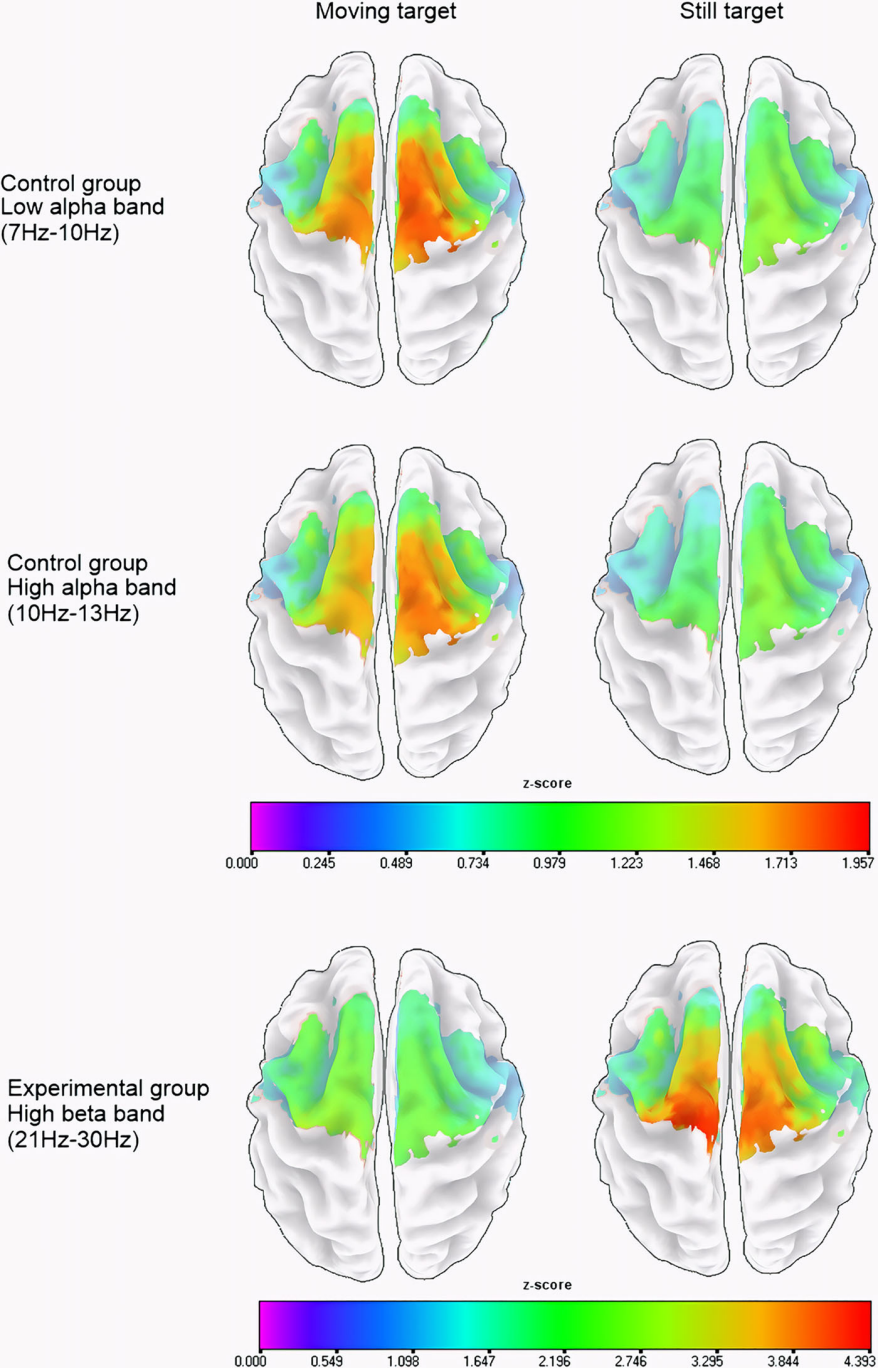
Cortical maps of source density (average *z*‐values) in the control and experimental groups with the non‐dominant/more affected leg during the still and moving target tasks in the frequency bands that presented a significant statistical difference between tasks.

The control group only presented significant differences between tasks with the non‐dominant leg, concretely in the low alpha band (1.94 ± 1.29 during the moving target task vs. 1.42 ± 0.67 during the still target task, *Z* = 23.000, *p* = 0.036, *r* = 0.542) and in the high alpha band (1.92 ± 1.33 vs. 1.37 ± 0.60, *Z* = 21.000, *p* = 0.027, *r* = 0.572) (Figure 8). Analogously, nearly significant differences were present for the theta band (2.74 ± 1.82 vs. 1.42 ± 0.67, *Z* = 29.000, *p* = 0.078, *r* = 0.455) and for the low beta band (3.31 ± 2.21 vs. 2.47 ± 1.10, *Z* = 26.000, *p* = 0.053, *r* = 0.499). For the dominant leg, no significant difference in SMA activation between the two tasks was found, although there is a tendency for more SMA activation in the still target task.

The experimental group showed a higher SMA activation with the more affected leg during the still target task. Concretely, a significant difference was found in the high beta band (3.53 ± 1.41 vs. 4.52 ± 2.01, *Z* = 85.000, *p* = 0.041, *r* = 0.545) (Figure 8). Similarly, a nearly significant difference was found in the mid beta band (1.12 ± 0.50 vs. 1.41 ± 0.57, *Z* = 82.000, *p* = 0.064, *r* = 0.495). No significant differences between tasks were present with the less affected leg. All the reported effect sizes are moderate to large.

### 3.4 Differences of Reaction Times Between Groups and Legs

Table 2 shows the average (and standard deviation) reaction times for group, task, and leg. Despite the control group showing a lower reaction time overall, regardless of the task and leg, the statistically significant differences between groups were found during the moving target task with the non‐dominant/more affected leg and during the still target task with the dominant/less‐affected leg. With respect to the within‐group differences, the reaction times with the non‐dominant/more affected leg showed higher than with the dominant/less affected leg in both groups in the still‐target task, although only significant for the control group. In the moving target task, the reaction times of both legs were comparable in both groups.

**TABLE 2:** Average (standard deviation) of the reaction times for each group, task, and leg and the corresponding statistics of the differences between groups and legs.

		Control	Experimental	Between‐group statistic
Moving target	Dominant/Less affected	2.09 (0.60) s	2.57 (0.87) s	U(30) = 147.000, *p* = 0.161
	Non‐Dominant/More affected	1.87 (0.60) s	2.49 (1.02) s	**U(30) = 160.000, *p* = 0.050, *r* = 0.366**
	Between‐leg statistic	*Z* = −1.193, *p* = 0.233	*Z* = −0.454, *p* = 0.650	
Still target	Dominant/Less affected	1.03 (0.65) s	1.83 (0.89) s	**U(29) = 164.500, *p* = 0.008, *r* = 0.482**
	Non‐Dominant/More affected	1.71 (0.80) s	2.31 (1.45) s	U(29) = 127.000, *p* = 0.354
	Between‐leg statistic	***Z* = −2.385, *p* = 0.017, *r* = 0.616**	*Z* = −1.319, *p* = 0.187	

## 4 Discussion

This study reveals altered patterns of motor cortex activation in a group of athletes with neurological disorders, particularly increased activation of the SMA. This finding suggests that the SMA plays a key role in motor control in individuals with neurological disorders and that there may be various compensatory strategies at the neuronal level to ensure that performance is not impaired. According to previous literature, it has been demonstrated that the SMA is involved in both motor planning and execution, with the pre‐SMA being more closely related to early planning processes and the SMA to the later stages of motor execution. It is essential to distinguish between immediate motor planning, typically occurring 1–2 s prior to movement (associated with readiness potentials or CNV), and the broader state associated with task anticipation. In this study, no EEG analysis was performed for the 20‐s period with the eyes closed. Therefore, the study results show cortical activity during the interval between the opening of the eyes and the execution of the movement, which corresponds more closely to the motor preparation processes that immediately precede the action.

In our experimental design, this extended period captures the maintenance of a preparatory set required to organize a complex technical gesture without prior visual stimuli, which transcends short‐term motor programming.

Activity in the different EEG frequency bands reflects various aspects of movement control. Alpha oscillations are typically associated with attention and cortical inhibition during movement preparation, whereas beta oscillations are more closely related to planning, sensorimotor integration, and the execution of the action. (Athanasiou et al. 2018). The differences observed across the frequency bands may suggest that athletes with neurological disorders could use modified neural mechanisms to facilitate the preparation and execution of movements.

In this study, one of the main findings observed is that there is significant activation of the SMA between limbs in the group of individuals with neurological disorders. There was greater activation of the SMA when using the affected limb compared to the less affected one, particularly in the high‐beta band. This pattern could indicate not only a deficit but also a greater demand for neural activation to achieve motor performance with the affected limb. This increase in cortical activation could be related to impaired communication between different cortical regions or to reduced neural processing, which may be consistent with previous studies linking neurological lesions to a reduction in processing speed (Boy et al. 2010). In the sports context, these alterations can result in decreased performance.

When comparing the groups, the group of athletes with neurological disorders showed greater bilateral activation of the SMA compared to the controls, particularly during the static target task. This finding is in line with previous studies describing greater SMA activation in individuals with neurological disorders during voluntary movement (Eckert et al. 2006; Foltys et al. 2003). These findings may reflect a compensatory mechanism, where additional cortical areas are recruited to maintain motor performance under impaired conditions (Caçola et al. 2016). In this sense, SMA overactivation could represent an adaptive response to functional deficits instead of a maladaptive process.

Consistent with our study, individuals with brain disorders have a greater requirement of the SMA, as they show bilateral activation of the SMA compared to the control group. The experimental groups resemble the control group by activating the contralateral SMA more, especially with the affected or non‐dominant leg. Research such as Mutha et al. suggests that the left hemisphere is specialized in motor planning and coordination, while the right hemisphere plays an important role in modifying ongoing actions (2012); this may relate to the athletes' automatization in the static target task. Activity in the SMA appears to be related to motor imagination, as evidenced in the study by Mizuguchi et al. (2012).

The difficulty and demands of the task itself appear to play a key role in the activation of the SMA. The control group showed significant differences depending on the task, particularly during the moving target condition, while the experimental group showed greater activation of the superior SMA during the static task.

This supports the idea that SMA activity is more influenced by the task context than by lateralization alone (Jenkins et al. 2000). In the static task, participants were asked to start the movement following a verbal command, which may increase dependence on internally generated motor planning processes. In contrast, tasks requiring continuous adjustment to external stimuli may involve different neural circuits, as suggested by Buetefisch et al. (2014). These findings reinforce the idea that SMA activation depends largely on the functional demands and complexity of the task.

In terms of motor performance, there were differences in reaction times depending on the group, the task, and the limb. The control group generally showed faster reaction times compared to the other group, which had slower reaction times, especially with the affected limb. These results are consistent with previous studies reporting slower reaction times in individuals with neurological impairments (Churchill et al. 2021; Dautricourt et al. 2017; Immink et al. 2021), as well as differences in reaction times between dominant and non‐dominant limbs (Janković et al. 2022). In the control group, reaction times were slower when using the non‐dominant limb, which also supports the role of motor dominance in performance.

Although both SMA activation and reaction time varied according to conditions, no correlation analysis was performed in this study. Therefore, it is not possible to establish a direct relationship between neural activity and behavioral performance. Previous studies have reported that stimulation or modulation of the SMA can influence motor control and movement performance (Mackenzie, 2015; Premji et al., 2011). In relation to athletes, one hypothesis we put forward is that stimulation in this area may decrease reaction times, which would lead to an improvement in sports performance. However, the current findings should be interpreted with caution in terms of this. Future studies should investigate the relationship between cortical activation patterns and behavioral outcomes to better understand their functional significance.

Overall, these findings suggest that athletes with neurological disorders show altered patterns of SMA activation that may reflect compensatory mechanisms designed to facilitate motor control. All of these cortical adaptations appear to be influenced by both the side of limb involvement and the complexity of the task, highlighting the complexity of motor control in neurological disorders.

While it is true that the number of tests increases the probability of Type I error, the family‐wise error rate (FWER) was not controlled here because it is not appropriate in this study, given that it rests on a logical fallacy thoroughly documented in the methodological literature. As Rubin (2021) and García‐Pérez (2023) demonstrate, FWER correction is only logically required for union‐intersection inference (“at least one test is significant”), not for separate individual inferences about distinct hypotheses; applying family‐based error rates to individual tests is an ecological fallacy, since the probability of a Type I error for each individual test remains at α = 0.05 regardless of how many other tests are conducted in the same study. The definition of what constitutes a family of tests is itself arbitrary and theoretically incoherent: as Perneger (1998) and Rothman (1990) argue, a finding's significance would change depending on how many unrelated tests appear in the same paper, which is scientifically nonsense. In this case, the six frequency bands analyzed here are not interchangeable replications of the same measurement—theta, alpha, and beta oscillations reflect physiologically distinct neural generators and functional processes (Cavanagh and Frank 2014; Klimesch et al. 2007; Pfurtscheller and Neuper 1994), and standard EEG practice treats them as separate hypothesis families (Maris and Oostenveld 2007; Keil et al., 2014). Applying blanket correction across all 48+ comparisons would reduce the effective alpha to approximately 0.001, catastrophically reducing statistical power in a small clinical sample (Button et al. 2013; Lieberman and Cunningham 2009), and would incur unacceptable Type II error costs in what is explicitly an exploratory study (Althouse 2016; Bender and Lange 2001). Nevertheless, the tendency is similar for all frequency bands when a significant result was found in the same “family” of tests and not in the others (which also often show inverse or no tendencies). Consequently, the conditions of the significant results are very likely pointing to actual findings rather than to Type I errors.

### 4.1 Limitations

A significant limitation of this study was the manual throwing of the ball during the moving target task; this may have affected reaction time measurements. Furthermore, although a 20‐s period with eyes closed was included prior to the movement, this interval was used to establish a baseline and was not included in the EEG analyses. Therefore, the results presented reflect cortical activity during the time interval between eye opening and the execution of the movement, which may involve both motor preparation and attentional and sensorimotor processes. Additionally, no movement quality metrics (e.g., trajectory, accuracy, and kinematics) were collected, making it difficult to interpret whether increased SMA activation reflects compensation or inefficiency. Another limitation of this study is the heterogeneity of the neurological conditions included in the experimental group. The participants had different etiologies, which may imply differences in lesion location, the degree of motor impairment, and cortical reorganization processes. Because the sample size was small, it was not possible to perform subgroup analyses or include clinical severity as a covariate. The results should be interpreted as an approximation of brain activation patterns in athletes with neurological disorders, rather than as specific characteristics of each condition. Another limitation is the mixed leg dominance in the experimental group, compared to all right‐leg dominant controls, which may affect “dominant versus affected” comparisons and hemispheric interpretations.

Although the significance of some of the results obtained exceeds the alpha level of 0.05, their *p*‐values remain low (<0.08) and reinforce the significant differences observed, as they follow the same trend in adjacent frequency bands, thereby corroborating the underlying differences detected.

We also acknowledge the limitations inherent to EEG source imaging with a 32‐channel system. While eLORETA achieves mathematically exact peak localization in noise‐free conditions and has been validated across a range of electrode densities, 32‐channel arrays operate below the spatial Nyquist rate of scalp EEG. As a consequence, and also considering that we did not use individual MRI to estimate the sources, the SMA activity reported here should be interpreted as a probabilistic source estimate rather than an anatomically precise localization: eLORETA's inherent spatial smoothing—a mathematical property of all weighted minimum norm inverse solutions—produces broad activation blobs spanning several centimeters, making it impossible to reliably distinguish adjacent medial frontal structures such as SMA, pre‐SMA, and dorsal ACC at this electrode density. Source leakage from neighboring generators cannot be excluded, and our regional interpretations should be understood as reflecting activity within a broad medial frontal cluster, mostly including the SMA, rather than a finely defined anatomical subregion. These constraints are common to low‐density EEG source imaging and do not invalidate the observed effects, which are consistent across subjects and statistically robust, but they do warrant caution against overly specific and anatomically accurate conclusions.

## 5 Conclusions

This study demonstrates altered motor cortical recruitment patterns in athletes with neurological disorders, characterized by increased SMA activation during tasks performed with the affected limb. These findings suggest that there may be compensatory neural strategies to facilitate motor control under altered neurological conditions. The results obtained regarding activation patterns between affected and unaffected limbs reinforce the role of the SMA in motor adaptation. From a clinical perspective, these results may contribute to a better understanding of the mechanisms underlying neuroplasticity and serve as a basis for specific neurorehabilitation approaches. These findings should be interpreted with caution given the relatively small sample size and the number of comparisons performed.

This study advances the understanding of supplementary motor area activation and cortical activity during tasks in athletes with and without neurological disorders. No previous study has addressed this topic using a similar approach, specifically combining an adapted motor task related to soccer with source‐level EEG analysis using eLORETA. The involvement of the SMA has been widely described in the previous literature on motor control and neurological disorders. The present study offers a novel perspective by examining its activation in athletes with neurological disorders in a sport‐specific context.

## Author Contributions

**Francisco Echevarría‐Lasaga**: Conceptualization, Methodology, Investigation, Writing – Original Draft, Writing – Review & Editing, Data Curation, Formal Analysis, **Ismael Sanz‐Esteban**: Conceptualization, Methodology, Writing – Review & Editing, Investigation, Supervision, Project Administration, **Cecilia Estrada‐Barranco**: Validation, Writing – Review & Editing, Supervision, Conceptualization, **Isabel Rodríguez‐Costa**: Writing – Review & Editing, Validation, Supervision, **Juan José García‐Hernández**: conceptualization, investigation, resources, methodology, writing – review & editing, project administration, **J. Ignacio Serrano**: Writing – Review & Editing, Investigation, Methodology, Formal Analysis, Software, Data Curation, Visualization

## Funding

The authors have nothing to report.

## Ethics Statement

The study was submitted to the Ethics Committee of the Hospital Clínico San Carlos, in accordance with the Declaration of Helsinki, with internal code 23/246‐E_TFM and to the Ethics Committee of the European University, obtaining approval to carry out the study in both places.

## Consent

All participants in the study signed an informed consent before their participation.

## Conflicts of Interest

The authors declare no conflicts of interest.

## Data Availability

Data will be available upon reasonable request.

## References

[brb371522-bib-0001] Althouse, A. D. 2016. “Adjust for Multiple Comparisons? It's Not That Simple.” The Annals of Thoracic Surgery 101, no. 5: 1644–1645. 10.1016/j.athoracsur.2015.11.024. 27106412

[brb371522-bib-0002] Athanasiou, A. , M. A. Klados , C. Styliadis , N. Foroglou , K. Polyzoidis , and P. D. Bamidis . 2018. “Investigating the Role of Alpha and Beta Rhythms in Functional Motor Networks.” Neuroscience 378: 54–70. 27241945 10.1016/j.neuroscience.2016.05.044

[brb371522-bib-0003] Badia, M. , M. B. Orgaz , M. A. Verdugo , A. M. Ullán , and M. Martínez . 2013. “Relationships Between Leisure Participation and Quality of Life of People With Developmental Disabilities.” Journal of Applied Research in Intellectual Disabilities 26, no. 6: 533–545. 10.1111/jar.12052. 23613480

[brb371522-bib-0004] Bender, R. , and S. Lange . 2001. “Adjusting for Multiple Testing—When and How?” Journal of Clinical Epidemiology 54, no. 4: 343–349. 10.1016/S0895-4356(00)00314-0. 11297884

[brb371522-bib-0005] Boy, F. , M. Husain , K. D. Singh , and P. Sumner . 2010. “Supplementary Motor Area Activations in Unconscious Inhibition of Voluntary Action.” Experimental Brain Research 206: 441–448. 10.1007/s00221-010-2417-x. 20871983

[brb371522-bib-0006] Buetefisch, C. M. , K. P. Revill , L. Shuster , B. Hines , and M. Parsons . 2014. “Motor Demand‐Dependent Activation of Ipsilateral Motor Cortex.” Journal of Neurophysiology 112, no. 4: 999–1009. 10.1152/jn.00110.2014. 24848477 PMC4122744

[brb371522-bib-0007] Button, K. S. , J. P. A. Ioannidis , C. Mokrysz , et al. 2013. “Power Failure: Why Small Sample Size Undermines the Reliability of Neuroscience.” Nature Reviews Neuroscience 14, no. 5: 365–376. 10.1038/nrn3475. 23571845

[brb371522-bib-0008] Caçola, P. , M. Ibana , M. Ricard , and C. Gabbard . 2016. “Children With Developmental Coordination Disorder Demonstrate a Spatial Mismatch When Estimating Coincident‐timing Ability With Tools.” Research in Developmental Disabilities 48: 124–131. 10.1016/j.ridd.2015.10.021. 26555384

[brb371522-bib-0009] Cañas, A. , M. Juncadella , R. Lau , A. Gabarrós , and M. Hernández . 2018. “Working Memory Deficits After Lesions Involving the Supplementary Motor Area.” Frontiers in Psychology 9: 765. 10.3389/fpsyg.2018.00765. 29875717 PMC5974158

[brb371522-bib-0010] Cavanagh, J. F. , and M. J. Frank . 2014. “Frontal Theta as a Mechanism for Cognitive Control.” Trends in Cognitive Sciences 18, no. 8: 414–421. 10.1016/j.tics.2014.04.012. 24835663 PMC4112145

[brb371522-bib-0011] Chang, C. Y. , S. H. Hsu , L. Pion‐Tonachini , and T. P. Jung . 2020. “Evaluation of Artifact Subspace Reconstruction for Automatic Artifact Components Removal in Multi‐Channel EEG Recordings.” IEEE Transactions on Biomedical Engineering 67, no. 4: 1114–1121. 10.1109/TBME.2019.2930186. 31329105

[brb371522-bib-0012] Churchill, N. W. , M. G. Hutchison , S. J. Graham , and T. A. Schweizer . 2021. “Brain Function Associated With Reaction Time After Sport‐Related Concussion.” Brain Imaging and Behavior 15: 1508–1517. 10.1007/s11682-020-00349-9. 32851585

[brb371522-bib-0013] Dautricourt, S. , I. Violante , E. J. Mallas , et al. 2017. “Reduced Information Processing Speed and Event‐Related EEG Synchronization in Traumatic Brain Injury.” Neurology 88: no. 16 (suppl): P6–149. 10.1212/WNL.88.16_supplement.P6.149.

[brb371522-bib-0014] Delorme, A. , and S. Makeig . 2004. “EEGLAB: an Open Source Toolbox for Analysis of Single‐Trial EEG Dynamics Including Independent Component Analysis.” Journal of Neuroscience Methods 134, no. 1: 9–21. 10.1016/j.jneumeth.2003.10.009. 15102499

[brb371522-bib-0015] Dinomais, M. , G. Lignon , E. Chinier , et al. 2014. “Effect of Motor Imagery in Children With Unilateral Cerebral Palsy: fMRI Study.” PLoS ONE 9, no. 4: e93378. 10.1371/journal.pone.0093378. 24718311 PMC3981713

[brb371522-bib-0016] Eckert, T. , T. Peschel , H. J. Heinze , and M. Rotte . 2006. “Increased Pre‐SMA Activation in Early PD Patients During Simple Self‐Initiated Hand Movements.” Journal of Neurology 253: 199–207. 10.1007/s00415-005-0956-z. 16222427

[brb371522-bib-0017] Feigin, V. L. , M. D. Abate , Y. H. Abate , et al. 2024. “Global, Regional, and National Burden of Stroke and Its Risk Factors, 1990–2021: A Systematic Analysis for the Global Burden of Disease Study 2021.” The Lancet Neurology 23, no. 10: 973–1003. 39304265 10.1016/S1474-4422(24)00369-7 PMC12254192

[brb371522-bib-0018] Ferree, T. C. 2006. “Spherical Splines and Average Referencing in Scalp Electroencephalography.” Brain Topography 19, no. 1–2: 43–52. 10.1007/s10548-006-0011-0. 17019635

[brb371522-bib-0019] Foltys, H. , T. Krings , I. G. Meister , et al. 2003. “Motor Representation in Patients Rapidly Recovering After Stroke: A Functional Magnetic Resonance Imaging and Transcranial Magnetic Stimulation Study.” Clinical Neurophysiology 114, no. 12: 2404–2415. 10.1016/s1388-2457(03)00263-3. 14652101

[brb371522-bib-0020] García‐Pérez, M. A. 2023. “Use and Misuse of Corrections for Multiple Testing.” Methods in Psychology 8: 100120. 10.1016/j.metip.2023.100120.

[brb371522-bib-0021] Hanrahan, S. J. 2015. “Psychological Skills Training for Athletes With Disabilities.” Australian Psychologist 50, no. 2: 102–105. 10.1111/ap.12083.

[brb371522-bib-0022] Hill, N. M. , and W. Schneider . 2006. “Brain Changes in the Development of Expertise: Neuroanatomical and Neurophysiological Evidence About Skill‐Based Adaptations.” In The Cambridge Handbook of Expertise and Expert Performance, edited by K. A. Ericsson N. Charness P. J. Feltovich , and R. R. Hoffman , 655–683. Cambridge University Press. 10.1017/CBO9780511816796.037.

[brb371522-bib-0023] Immink, M. A. , M. Pointon , D. L. Wright , and F. E. Marino . 2021. “Prefrontal Cortex Activation During Motor Sequence Learning Under Interleaved and Repetitive Practice: A Two‐Channel Near‐Infrared Spectroscopy Study.” Frontiers in Human Neuroscience 15: 644968. 10.3389/fnhum.2021.644968. 34054448 PMC8160091

[brb371522-bib-0024] Jaeger, L. , L. Marchal‐Crespo , P. Wolf , R. Riener , L. Michels , and S. Kollias . 2014. “Brain Activation Associated With Active and Passive Lower Limb Stepping.” Frontiers in Human Neuroscience 8: 828. 10.3389/fnhum.2014.00828. 25389396 PMC4211402

[brb371522-bib-0025] Janković, D. , A. Čvorović , M. Dopsaj , I. Prćić , and F. Kukić . 2022. “Effects of the Task Complexity on the Single Movement Response Time of Upper and Lower Limbs in Police Officers.” International Journal of Environmental Research and Public Health 19, no. 14: 8695. 10.3390/ijerph19148695. 35886550 PMC9321739

[brb371522-bib-0026] Jenkins, I. H. , M. Jahanshahi , M. Jueptner , R. E. Passingham , and D. J. Brooks . 2000. “Self‐Initiated versus Externally Triggered Movements: II. The Effect of Movement Predictability on Regional Cerebral Blood Flow.” Brain 123, no. 6: 1216–1228. 10.1093/brain/123.6.1216. 10825359

[brb371522-bib-0027] Kapreli, E. , S. Athanasopoulos , M. Papathanasiou , et al. 2006. “Lateralization of Brain Activity During Lower Limb Joints Movement: An fMRI Study.” NeuroImage 32, no. 4: 1709–1721. 10.1016/j.neuroimage.2006.05.043. 16859927

[brb371522-bib-0028] Keil, A. , S. Debener , G. Gratton , et al. 2014. “Committee Report: Publication Guidelines and Recommendations for Studies Using Electroencephalography and Magnetoencephalography.” Psychophysiology 51, no. 1: 1–21. 10.1111/psyp.12147. 24147581

[brb371522-bib-0029] Klimesch, W. , P. Sauseng , and S. Hanslmayr . 2007. “EEG Alpha Oscillations: The Inhibition‐Timing Hypothesis.” Brain Research Reviews 53, no. 1: 63–88. 10.1016/j.brainresrev.2006.06.003. 16887192

[brb371522-bib-0031] Lieberman, M. D. , and W. A. Cunningham . 2009. “Type I and Type II Error Concerns in fMRI Research: Re‐Balancing the Scale.” Social Cognitive and Affective Neuroscience 4, no. 4: 423–428. 10.1093/scan/nsp052. 20035017 PMC2799956

[brb371522-bib-0032] Liu, H. , W. Cai , L. Xu , W. Li , and W. Qin . 2020. “Differential Reorganization of SMA Subregions After Stroke.” Frontiers in Human Neuroscience 13: 468. 10.3389/fnhum.2019.00468. 32184712 PMC7059000

[brb371522-bib-0033] Mackenzie, T. 2015. “The Influence of Area 5 on the Excitation of Primary Motor Cortex.” Master's thesis, McMaster University. Retrieved from https://prod‐ms‐be.lib.mcmaster.ca/server/api/core/bitstreams/0dfdd00c‐ec52‐4421‐8735‐921175943a20/content.

[brb371522-bib-0034] Mancuso, L. , T. Costa , A. Nani , et al. 2019. “The Homotopic Connectivity of the Functional Brain: A Meta‐Analytic Approach.” Scientific Reports 9: 3346. 10.1038/s41598-019-40188-3. 30833662 PMC6399443

[brb371522-bib-0035] Maris, E. , and R. Oostenveld . 2007. “Nonparametric Statistical Testing of EEG‐ and MEG‐Data.” Journal of Neuroscience Methods 164, no. 1: 177–190. 10.1016/j.jneumeth.2007.03.024. 17517438

[brb371522-bib-0036] Mizuguchi, N. , H. Nakata , Y. Uchida , and K. Kanosue . 2012. “Motor Imagery and Sport Performance.” The Journal of Physical Fitness and Sports Medicine 1, no. 1: 103–111. 10.7600/jpfsm.1.103.

[brb371522-bib-0037] Mutha, P. K. , K. Y. Haaland , and R. L. Sainburg . 2012. “The Effects of Brain Lateralization on Motor Control and Adaptation.” Journal of Motor Behavior 44, no. 6: 455–469. 10.1080/00222895.2012.747482. 23237468 PMC3549328

[brb371522-bib-0038] Nachev, P. , C. Kennard , and M. Husain . 2008. “Functional Role of the Supplementary and Pre‐supplementary Motor Areas.” Nature Reviews Neuroscience 9, no. 11: 856–869. 10.1038/nrn2478. 18843271

[brb371522-bib-0039] Newton, J. M. , Y. Dong , J. Hidler , et al. 2008. “Reliable Assessment of Lower Limb Motor Representations With fMRI.” NeuroImage 43, no. 1: 136–146. 10.1016/j.neuroimage.2008.06.043. 18675363 PMC2658811

[brb371522-bib-0040] Ortega Suero, G. , M. J. Abenza Abildúa , C. Serrano Munuera , et al. 2023. “Epidemiology of Ataxia and Hereditary Spastic Paraplegia in Spain: A Cross‐Sectional Study.” Neurología (English Edition) 38, no. 6: 379–386. . 10.1016/j.nrleng.2023.04.003. 37120112

[brb371522-bib-0041] Pérez Tejero, J. , R. Reina Vaíllo , and D. Sanz Rivas . 2012. “Adapted Physical Activity for people with disability in Spain: scientific perspectives and current issuess.” Cultura_Ciencia_Deporte [CCD] 7, no. 21: 213–224. 10.12800/ccd.v7i21.86.

[brb371522-bib-0042] Pérez‐Tejero, J. , and M. D. C. Ocete‐Calvo . 2018. “Personas con Discapacidad y Práctica Deportiva en España.” In El libro blanco para Personas con Discapacidad en España, edited by N. Mendoza , R. Reina , D. Sanz , and J. Pérez‐Trejo , 55–77. Ediciones Cinca.

[brb371522-bib-0043] Pascual‐Marqui, R. D. , D. Lehmann , M. Koukkou , et al. 2011. “Assessing Interactions in the Brain With Exact Low‐Resolution Electromagnetic Tomography.” Philosophical Transactions of the Royal Society A: Mathematical, Physical and Engineering Sciences 369, no. 1952: 3768–3784. 10.1098/rsta.2011.0081 21893527

[brb371522-bib-0044] Perneger, T. V. 1998. “What's Wrong With Bonferroni Adjustments.” BMJ 316, no. 7139: 1236–1238. 10.1136/bmj.316.7139.1236. 9553006 PMC1112991

[brb371522-bib-0045] Pfurtscheller, G. , and C. Neuper . 1994. “Event‐Related Synchronization of Mu Rhythm in the EEG Over the Cortical Hand Area in Man.” Neuroscience Letters 174, no. 1: 93–96. 10.1016/0304-3940(94)90127-9. 7970165

[brb371522-bib-0046] Pion‐Tonachini, L. , K. Kreutz‐Delgado , and S. Makeig . 2019. “ICLabel: An Automated Electroencephalographic Independent Component Classifier, Dataset, and Website.” NeuroImage 198: 181–197. 10.1016/j.neuroimage.2019.05.026. 31103785 PMC6592775

[brb371522-bib-0047] Potgieser, A. R. , B. M. de Jong , M. Wagemakers , E. W. Hoving , and R. J. Groen . 2014. “Insights From the Supplementary Motor Area Syndrome in Balancing Movement Initiation and Inhibition.” Frontiers in Human Neuroscience 8: 960. 25506324 10.3389/fnhum.2014.00960 PMC4246659

[brb371522-bib-0048] Premji, A. , N. Rai , and A. Nelson . 2011. “Area 5 Influences Excitability Within the Primary Motor Cortex in Humans.” PLoS ONE 6, no. 5: e20023. 10.1371/journal.pone.0020023. 21603571 PMC3095637

[brb371522-bib-0049] Rodríguez‐Bazurto, J. V. , and E. K. Aguilar‐Morocho . 2021. “Importance of Soccer Practice for the Development of Coordinative Capacities.” Domain Science 7, no. 6: 475–492. 10.23857/dc.v7i6.2344.

[brb371522-bib-0050] Rothman, K. J. 1990. “No Adjustments Are Needed for Multiple Comparisons.” Epidemiology 1, no. 1: 43–46. 10.1097/00001648-199001000-00010. 2081237

[brb371522-bib-0051] Rubin, M. 2021. “When to Adjust Alpha During Multiple Testing: A Consideration of Disjunction, Conjunction, and Individual Testing.” Synthese 199, no. 3–4: 10969–11000. 10.1007/s11229-021-03276-4.

[brb371522-bib-0052] Ruddy, K. L. , A. Leemans , and R. G. Carson . 2017. “Transcallosal Connectivity of the Human Cortical Motor Network.” Brain Structure and Function 222, no. 3: 1243–1252. 10.1007/s00429-016-1274-1. 27469272 PMC5368198

[brb371522-bib-0053] Sahyoun, C. , A. Floyer‐Lea , H. Johansen‐Berg , and P. M. Matthews . 2004. “Towards an Understanding of Gait Control: Brain Activation During the Anticipation, Preparation and Execution of Foot Movements.” NeuroImage 21, no. 2: 568–575. 10.1016/j.neuroimage.2003.09.065. 14980558

[brb371522-bib-0054] Shirinbayan, S. I. , A. M. Dreyer , and J. W. Rieger . 2019. “Cortical and Subcortical Areas Involved in the Regulation of Reach Movement Speed in the Human Brain: An fMRI Study.” Human Brain Mapping 40, no. 1: 151–162. 10.1002/hbm.24361. 30251771 PMC6865438

[brb371522-bib-0055] Takakusaki, K. 2017. “Functional Neuroanatomy for Posture and Gait Control.” Journal of Movement Disorders 10, no. 1: 1–17. 10.14802/jmd.16062. 28122432 PMC5288669

[brb371522-bib-0056] Turner‐Stokes, L. , N. Sykes , and E. Silber . 2008. “Long‐Term Neurological Conditions: Management at the Interface Between Neurology, Rehabilitation and Palliative Care.” Clinical Medicine 8, no. 2: 186–191. 18478869 10.7861/clinmedicine.8-2-186 PMC4953006

[brb371522-bib-0057] Welniarz, Q. , Gallea , J. C. Lamy , et al. 2019. “The Supplementary Motor Area Modulates Interhemispheric Interactions During Movement Preparation.” Human Brain Mapping 40, no. 7: 2125–2142. 10.1002/hbm.24512. 30653778 PMC6865634

[brb371522-bib-0058] Wright, M. J. , D. T. Bishop , R. C. Jackson , and B. Abernethy . 2010. “Functional MRI Reveals Expert–Novice Differences During Sport‐Related Anticipation.” Neuroreport 21, no. 2: 94–98. 10.1097/WNR.0b013e328333dff2. 20051784

[brb371522-bib-0059] Yin, J. , K. Yang , C. Zhou , and J. Dang . 2025. “Motor Expertise Modulates Cortical Activation During Imagery of Simple and Complex Actions.” Proceedings of the National Academy of Sciences 122, no. 39: e2515027122. 10.1073/pnas.2515027122. PMC12501113 40982676

[brb371522-bib-0060] Zhang, L. , F. Qiu , H. Zhu , M. Xiang , and L. Zhou . 2019. “Neural Efficiency and Acquired Motor Skills: An fMRI Study of Expert Athletes.” Frontiers in Psychology 10: 2752. 10.3389/fpsyg.2019.02752. 31866917 PMC6908492

